# Resolution of inflammation: an integrated view

**DOI:** 10.1002/emmm.201202382

**Published:** 2013-04-17

**Authors:** Almudena Ortega-Gómez, Mauro Perretti, Oliver Soehnlein

**Affiliations:** 1 Institute for Cardiovascular Prevention, LMUMunich, Germany; 2 William Harvey Research Institute, Barts and The London School of MedicineLondon, UK; 3 Department of Pathology, AMCAmsterdam, The Netherlands

**Keywords:** apoptosis, efferocytosis, macrophage reprogramming, pro-resolving drugs, tissue homeostasis

## Abstract

Resolution of inflammation is a coordinated and active process aimed at restoration of tissue integrity and function. This review integrates the key molecular and cellular mechanisms of resolution. We describe how abrogation of chemokine signalling blocks continued neutrophil tissue infiltration and how apoptotic neutrophils attract monocytes and macrophages to induce their clearance. Uptake of apoptotic neutrophils by macrophages reprograms macrophages towards a resolving phenotype, a key event to restore tissue homeostasis. Finally, we highlight the therapeutic potential that derives from understanding the mechanisms of resolution.

## Introduction

**Figure 1:**
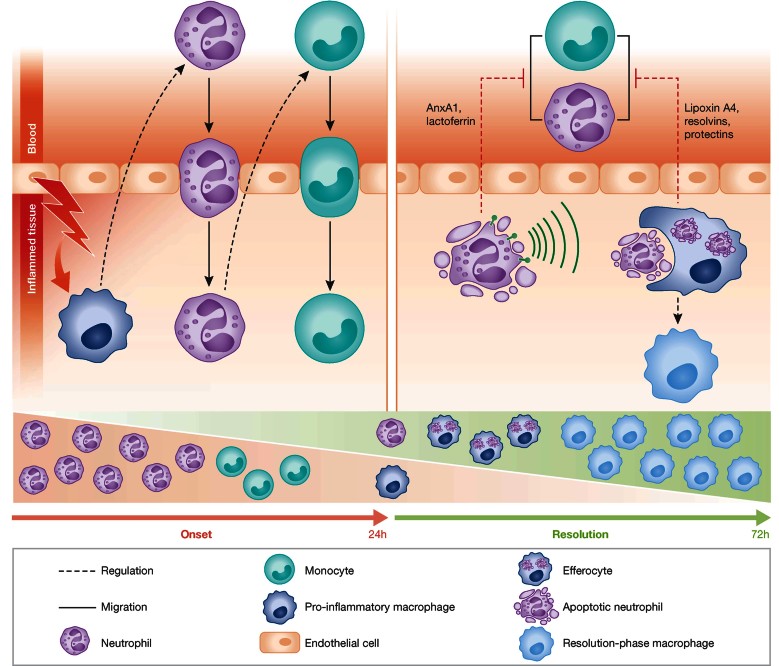
Cellular interplay during resolution of inflammation. Overview of cellular processes during onset (left) and resolution (right) of inflammation. During early phases of inflammation tissue-resident cells sense damage and launch the release of signals that induce rapid neutrophil and delayed monocyte emigration. Resolution is initiated when neutrophils become apoptotic thus secreting mediators that inhibit continued neutrophil infiltration. Ingestion of apoptotic neutrophils changes the macrophage phenotype towards a resolution-phase macrophage, which promotes return to tissue homeostasis. A switch in tissue (stromal) cells can also contribute to generate the initial signals for resolution to start.

Inflammation is a pathophysiological response to infection or tissue damage. In order to neutralize the causing agent, the innate immune system launches a program that unfolds in several phases (Soehnlein & Lindbom, 2010). Initially, tissue-resident cells of the innate immune system detect the damaging insult and alarm circulating neutrophils. These migrate to the inflamed tissue, promote recruitment of inflammatory monocytes and potentiate the pro-inflammatory environment allowing to appropriately deal with the inflammogen (Mantovani et al, 2011). Under normal conditions, neutrophils undergo apoptosis after performing their action at the inflamed site (Fox et al, 2010) and macrophages ingest apoptotic neutrophils. Clearance of apoptotic neutrophils prompts a switch from a pro- to an anti-inflammatory macrophage phenotype (Fadok et al, 1998; Michlewska et al, 2009), which is a prerequisite for macrophage egress via the lymphatic vessels favouring return to tissue homeostasis (Fig 1).

The past few years have witnessed an appreciation of the complex resolution of inflammation process, characterized by molecular and cellular events that ultimately assure tissue repair and regain of physiological function. A crucial point is the concept that resolution is an active phenomenon, aimed at actively suppressing and extinguishing a vibrant inflammatory reaction once its main objective (*e.g.* clearance of bacteria) is attained. In this review we highlight the essential mechanisms underlying resolution of inflammation and discuss how these mechanisms can be targeted to accelerate return to tissue integrity.

## Chemokine depletion sets the brakes on neutrophil tissue infiltration

**Figure 2:**
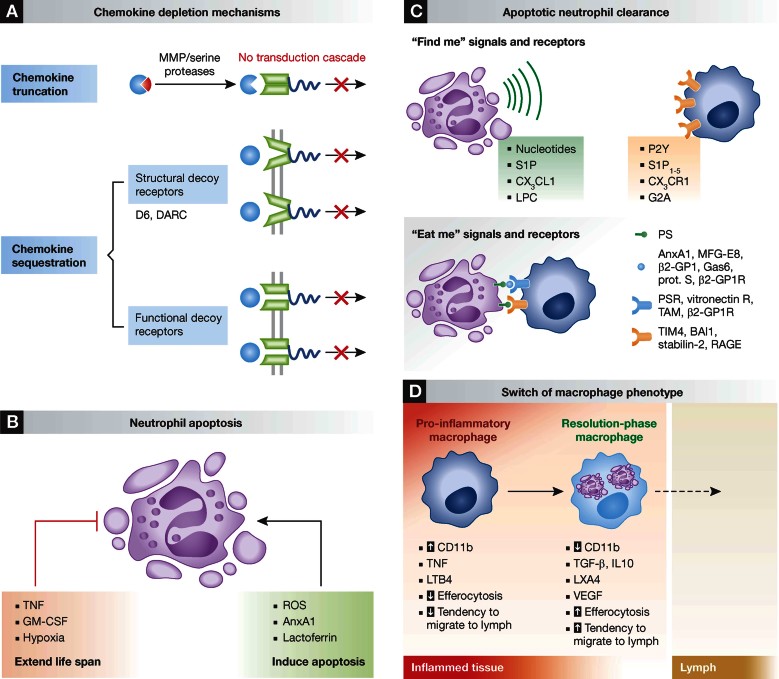
Mechanisms of neutrophil- and macrophage-driven resolution. A. Depletion of chemokines during resolution. MMPs cleave CC and CXC chemokines rendering them non-functional. Structural decoy receptors such as D6 and DARC sequester chemokines without subsequent signal transduction. Functional decoy receptors are classical chemokine receptors with repressed signalling. B. Factors controlling neutrophil life span at sites of inflammation. Interestingly, the pro-apoptotic stimuli can often override those that augment neutrophil life span. C. Upon apoptosis *find me* signals such as nucleotides, S1P, CX_3_CL1 and LPC are released that attract scavengers. These recognize apoptotic cells via *eat me* signals exposed on the cell surface. Clearance is mediated by direct cell–cell contact or by involvement of bridging molecules. D. In response to local mediators and upon efferocytosis, pro-inflammatory macrophages switch to resolution-phase macrophages.

Chemokines are molecular cues that orchestrate leukocyte migration to sites of inflammation. Abrogation of neutrophil influx is a prerequisite for resolution of inflammation and mechanisms such as chemokine cleavage by proteolysis and chemokine sequestration are necessary to attain a resolving environment (Fig 2A). Macrophage-specific MMP12 cleaves CXC chemokines in the ELR motif, which is crucial for receptor binding, thus rendering them unable to recruit neutrophils (Dean et al, 2008). MMP-dependent chemokine cleavage also depletes CC-chemokines that preferentially attract classical monocytes. For instance, cleaved CCL7 continues to bind CCR1, CCR2 and CCR3, but fails to induce downstream signalling and chemotaxis, thus acting as a general antagonist dampening inflammation (McQuibban et al, 2000, 2002).

Chemokine receptors possess a conserved DRY motif in the second intracellular loop which is involved in coupling to G-proteins. Receptors lacking the DRY motif sequester chemokines without launching a signalling cascade and are hence termed decoy receptors (Mantovani et al, 2006). Prominent members of this subfamily are duffy antigen receptor for chemokines (DARC) and D6. DARC is predominantly expressed by endothelial cells near sites of leukocyte extravasation and can sequester CC and CXC pro-inflammatory chemokines (Gardner et al, 2004). The lack of this atypical chemokine receptor leads to increased neutrophil influx into lung and liver upon application of lipopolysaccharide (LPS; Dawson et al, 2000; Luo et al, 2000) hence supporting its importance in termination of neutrophil recruitment. D6 on the other hand, binds a broad range of CC chemokines including most agonists of CCR1, CCR2 and CCR5 (Bonecchi et al, 2004). D6-deficient mice exhibit a dysregulated inflammatory reaction with impaired clearance of chemokines, resulting in tissue accumulation of neutrophils (Jamieson et al, 2005). After myocardial infarction, D6 prevents excessive infiltration of classical monocytes and neutrophils by scavenging CCL2 (Cochain et al, 2012) a phenomenon functionally associated with adverse remodelling and reduced ejection fraction. Another atypical chemokine receptor, CCX-CKR, scavenges constitutive chemokines such as CCL19, CCL21 and CCL25 which control homeostatic leukocyte trafficking. Therefore, an overriding function of this receptor in the resolution of inflammation appears rather unlikely (Bunting et al, 2013).

A sophisticated mechanism for chemokine entrapment is the generation of functional decoy receptors from classical chemokine receptors. In an environment rich in microbial peptides or pro-inflammatory cytokines, IL10 inhibits down-regulation of CCR1, CCR2 and CCR5, but these receptors are now unable to translate ligand binding into cell migration, thus trapping pro-inflammatory chemokines (D'Amico et al, 2000). A similar mechanism is centred on the up-regulation of CCR5 on apoptotic neutrophils leading to depletion of pro-inflammatory chemokines during the resolution phase of acute murine peritonitis (Ariel et al, 2006).

## Neutrophil apoptosis is central to resolution

Aborted neutrophil recruitment is but one of the steps required to reconstitute tissue homeostasis, clearance of neutrophils must also be achieved in due time. Despite the recent controversy regarding the life span of neutrophils (Dixon et al, 2012; Pillay et al, 2010), they are in general considered to be short-lived. Once emigrated, the lifespan of neutrophils can be modified by the local inflammatory environment (Fox et al, 2010; Geering & Simon, 2011; Fig 2B). For instance, macrophages secrete death receptor ligands such as Fas-ligand and TNF to promote neutrophil survival at low concentrations and neutrophil death at higher concentrations (van den Berg et al, 2001). In this regulatory pathway, phosphoinositide 3-kinase (PI3K)-mediated reactive oxygen species (ROS) production represents the key event in limiting cell survival and triggering apoptosis (Geering et al, 2011). Furthermore, Btk, a negative regulator of NADPH activation, is associated with neutrophil survival. Thus, Btk-deficient neutrophils feature high levels of ROS production, driven by excessive NADPH and PI3K activation (Honda et al, 2012). Other environmental signals such as hypoxia extend neutrophil lifespan; neutrophilic inflammation occurs in hypoxic environments, where hypoxia-inducible factor-1α (HIF1α) interferes with neutrophil survival (Walmsley et al, 2011). Finally, GM-CSF, a survival factor for neutrophils, triggers ERK activation and interference with ERK or other life span prolonging signalling pathways may allow for induction of neutrophil apoptosis.

Intriguingly, apoptotic neutrophils may attenuate inflammation through distinct mechanisms. Dying neutrophils secrete mediators that inhibit further neutrophil recruitment. One is annexin A1 (AnxA1; 37 kDa), a protein abundant in the cytosol of resting neutrophils. AnxA1 translocates to the plasma membrane when activated, where it interacts with the formyl peptide receptor 2 (FPR2/ALX) to moderate leukocyte adhesion and migration (Dalli et al, 2008; Perretti et al, 2002). It also promotes neutrophil apoptosis and clearance of dead neutrophils by macrophages (Perretti & Solito, 2004; Scannell et al, 2007). Another neutrophil-derived protein with similar activities is lactoferrin. It is contained within the secondary granules of neutrophils and when released, lactoferrin binds to specific receptors that trigger MAPK-mediated intracellular signalling, which is crucial in the regulation of cytoskeletal remodelling and cell adhesion (Bournazou et al, 2009). In a model of acute lung injury, lactoferrin application prevents neutrophil tissue infiltration, oedema formation and improves lung function (Table 1; Li et al, 2012). Nonetheless, lactoferrin exerts survival effects on neutrophils in rheumatoid arthritis (Wong et al, 2009) depending on its iron saturation level (Francis et al, 2011). Thus, the survival/apoptosis balance in neutrophils is monitored by an intricate and complex network of signalling factors which might present opposite effects depending on their concentration (*e.g.* TNF) or iron saturation state (*e.g.* lactoferrin).

**Table 1:** *In vivo* approaches to induce resolution of inflammation

Disease model	Drug	Effect on resolution	Refs.
Inhibition of leukocyte recruitment			
Air pouch model in mice	AnxA1 of human neutrophil-derived microparticles	Decrease of leukocyte infiltration	Dalli et al (2008)
Rheumatoid arthritis in mice	LXA4	Decrease of oedema, neutrophil influx, and mRNA levels of CXCL1, LTB4, and TNF after LXA4 treatment	Conte et al (2010)
Peritonitis in mice	Nanoparticles containing aspirin-triggered RvD1 or a lipoxinA_4_ analogue	Reduced neutrophil influx into the peritoneum, and resolution intervals	Norling et al (2011)
LPS-induced ALI	lactoferrin	Reduced lung neutrophil infiltration with less oedema and plasma leakage	Li et al (2012)
Air pouch and skin inflammation in mice	LXA4 analogue	Reduced neutrophil infiltration	Clish et al (1999)
Peritonitis in mice	RvE1	Reduced leukocyte infiltration into the peritoneum	Arita et al (2005)
Colitis in mice	RvE1	Increased survival rates, sustained body weight, improvement of histologic scores, reduced serum anti-2,4,6-trinitrobenzene sulfonic acid IgG and reduction of leukocyte infiltration and pro-inflammatory gene expression	Arita et al (2005)
Peritonitis in mice	Chemerin C15	Reduction of neutrophil and monocyte infiltration into the peritoneum, enhancement of apoptotic cell phagocytosis of by macrophages and reduction of pro-inflammatory mediators	Cash et al (2008)
Induction of neutrophil apoptosis			
Acute pleurisy, lung injury and arthritis in mice	R-roscovitine (CDK inhibitor)	Reduced presence of inflammatory cells (neutrophil and monocytes/macrophages) and amelioration of disease according to clinical scores	Rossi et al (2006)
Acute lung injury mice	15-epi-LXA4	Increased neutrophil apoptosis and efferocytosis	El Kebir et al (2009)
Lung inflammation in rats	Ectoine	Restoration of normal neutrophil apoptosis rates	(Sydlik et al 2013)
Pleurisy in rats	PD98059 (ERK inhibitor)	Decrease of total number macrophages and neutrophils in the pleural cavity, increased rate of neutrophil apoptosis	Sawatzky et al (2006)
Acute pleurisy in mice	Ac2-26 (AnxA1-active N-terminal peptide)	Reduction of neutrophils by induction of neutrophil apoptosis	Vago et al (2012)
Peritonitis in mice	Histone deacetylase inhibitors	AnxA1-dependent decrease in neutrophil, monocyte and macrophage infiltration	Montero-Melendez et al (2013)
Enhancement of efferocytosis and macrophage reprogramming			
Microbial peritonitis/air pouch model in mice	RvD1, RvD5, PD1s	Enhancement of bacterial killing, neutrophil efferocytosis and prevention of hypothermia. Acceleration of resolution in combination with antibiotics	Chiang et al (2012)
Allergic airway inflammation model in mice	RvE1	Suppression of the production of IL17, IL23 and IL6 and increased concentration of IFN-γ and LXA4 in brocheoalveolar lavage fluid, decrease of resolution intervals, acceleration of resolution of allergic airway pathology and hyper-responsiveness	Haworth et al (2008)
Sepsis model in rats	LXA4	Increase of animal survival, decrease of blood bacterial load and pro-inflammatory mediators	Walker et al (2011)
Peritonitis in mice	RGD-AnxA5	Enhancement of engulfment of apoptotic cells by macrophages and increase of secretion of IL-10 during efferocytosis	Schutters et al (2013)
CGD in mice	IFN-γ	Enhanced engulfment of apoptotic cells by macrophages	Fernandez-Boyanapalli et al (2010)
Peritonitis in CGD mice	IFN-γ	Enhanced engulfment of apoptotic cells by macrophages	Fernandez-Boyanapalli et al (2010)
Early renal fibrosis in rats	LXA4/benzoLXA4	Prevention of collagen deposition and renal apoptosis. Inhibition of TNF production and stimulation of IL-10	Borgeson et al (2011)
CGD in mice	IL4	Normalized CGD macrophage efferocytosis	Fernandez-Boyanapalli et al (2009)
CGD in mice	PS	Restored IL4-dependent macrophage reprogramming and efferocytosis	Fernandez-Boyanapalli et al (2009)
Stimulation of tissue repair			
Mucosal injury and ulceration in mice	AnxA1	Promotion of intestinal epithelial migration trough activation of FPR1-, Rac1- and NOX1-dependent redox signalling to induce wound repair	Leoni et al (2013)
Rheumatoid arthritis model in mice	Flavopiridol (CDK inhibitor)	Suppression of synovial hyperplasia and joint destruction	Sekine et al (2008)
Rheumatoid arthritis model in mice	CDK4/6-selective inhibitor	Suppression of synovial hyperplasia and joint destruction	Sekine et al (2008)
Peritoneal and lung inflammation in mice	PS/PC liposomes	Induction of TGF-β secretion, resulting in accelerated resolution of inflammation	Huynh et al (2002)
Myocardial infarction in rats	PS/PC liposomes	Induction of angiogenesis, preservation of small scars, prevention of ventricular dilatation and remodelling	Harel-Adar et al (2011)
Retinal ischaemia in mice	PS/PC liposomes	Reduced expression of pro-inflammatory genes, reduced neuronal death in the retina after ischaemia-reperfusion	Dvoriantchikova et al (2009)
Liver cirrhosis in rats	Vitamin A-coupled liposomes carrying siRNAgp46	Decrease in collagen recreation from HS cells and abrogation of hepatic stellate cells in fibrotic tissue through apoptosis, resulting in reduced fibrotic area in rat kidneys	Sato et al (2008)

ALI, acute lung injury; Anx, annexin; CGD, chronic glomerular disease; LXA4, IFN, interferon; Lipoxin A4; PC, phosphatidylcholine; PS, phosphatidlyserine; Rv, resolving.

Apoptotic neutrophils are cleared by macrophages via efferocytosis. Apoptotic neutrophils promote their own clearance by expressing *find me* and *eat me* signals (Fig 2C). *Find me* signals are secreted factors that attract scavengers. To date, four major *find me* signals have been described: lysophosphatidylcholine (LPC), sphingosine 1-phosphate (S1P), fractalkine (CX_3_CL1), and the nucleotides ATP and UTP (Elliott et al, 2009; Gude et al, 2008; Lauber et al, 2003; Truman et al, 2008). *Find me* signal gradients guide the efferocyte towards the dead cell through the involvement of G2A, S1P_1-5_, CX_3_CR1, and P2Y2 receptors, respectively. *Eat me* signals are surface markers which allow the identification of a dying cell. These signals can be either molecules exposed *de novo* at the cell membrane or existing ones that undergo modifications during apoptosis, as for instance peptides derived from the AnxA1 N-terminal domain (Blume et al, 2012). Phosphatidylserine (PS) on the outer membrane of the apoptotic cell is the best-known *eat me* signal. Direct recognition of PS by the efferocyte is mediated by a variety of receptors including TIM4, BAI1, stabilin-2 and the receptor for advanced glycation end products (RAGE; He et al, 2011; Ravichandran, 2011). In contrast, other receptors are engaged via soluble bridging molecules, one example being AnxA1 that links PS to the PS receptor (Arur et al, 2003). Similarly, MFG-E8 links PS and the vitronectin receptor, Gas 6 or Protein S facilitate contact between PS and TAM receptors, and β2-glycoprotein-I with its receptor and PS (Stitt et al, 1995). How these various receptors signal intracellularly to mediate cytoskeletal rearrangement thereby facilitating corpse uptake remains to be understood.

## A functional macrophage switch governs the return to tissue homeostasis

Macrophages possess a striking functional and phenotypic plasticity that becomes apparent during the resolution phase of inflammation. Upon apoptotic cell efferocytosis, macrophages turn off production of pro-inflammatory cytokines and lipid mediators and launch an anti-inflammatory transcriptional program characterized by the release of IL10 and TGF-β (Fadok et al, 1998; Fig 2D), quite reminiscent of the M2 alternative macrophage activation pattern. Transcriptomic analyses of murine resolution-phase macrophages shed light on the main features of macrophages during resolution *in vivo* (Stables et al, 2011). Resolution-phase macrophages are rich in molecules important for antigen processing and presentation and secrete T- and B-cell chemoattractants (XCL1, CCL5 and CXCL13). Consequently, the lymphocytes that repopulate sites of resolving inflammation comprise B1, NK, γδT, CD4^+^CD25^+^ and B2 cells which might exert a protective effect in the post-resolution phase (Rajakariar et al, 2008). In addition, resolution-phase macrophages express TIM4 and TGF-β, key molecules in the clearance of inflammatory cells and the return to tissue homeostasis. Functional characterization of resolving macrophages revealed lower levels of CD11b, enhanced capacity to engulf dead neutrophils, reduced responsiveness to TLR4 ligands, thus possibly leading to a “satiated” state with the ultimate departure through the lymph (Schif-Zuck et al, 2011). Clearly, the latter two studies indicate heterogeneity of resolution-phase macrophages and call for a standardization of the classification criteria. A chemokine scavenging-independent role for D6 in promoting macrophage-mediated resolution has recently been outlined. D6-deficient macrophages display a defect in conversion to a resolution phase macrophage. Furthermore, senescent human neutrophils and resolution-phase murine neutrophils present an increased expression of D6, suggesting that D6 might play a role in the interactions of apoptotic neutrophils with macrophages, as well as satiation of these macrophages (Pashover-Schallinger et al, 2012).

In addition, rapidly generated lipid mediators play a key role in orchestration of inflammation and its resolution (Serhan et al, 2008). In particular, the arachidonic acid pathway synthesizes pro-inflammatory lipids such as prostaglandin (PG) E2 and D2, and, during the resolution phase, pro-resolving bioactive lipid mediators including lipoxins and the ω3-unsaturated fatty acid-derivatives termed resolvins and protectins. Intriguingly, differential gene regulation of arachidonate metabolism-related enzymes has been reported in M1- and M2-polarized human macrophages (Martinez et al, 2006). M1 macrophages display a marked induction of COX2, with down-regulation of COX1, leukotriene A4 hydrolase, thromboxane A synthase 1 and arachidonate 5-lipoxygenase. Conversely, M2 macrophages show up-regulation of arachidonate 15-lipoxygenase and COX1. Furthermore, microsomal PGE synthase, the key enzyme in PGE2 production, is induced in macrophages by inflammatory M1 signals such as LPS and is functionally coupled to COX2 expression. In contrast, M2 stimuli such as IL4 and IL13 down-regulate mPGES expression in macrophages.

Functionally, lipoxin A4 (LXA4) inhibits neutrophil entry to tissue sites while promoting monocyte migration (Maddox et al, 1997). Cell-type specific signalling pathways downstream of FPR2/ALX activation by LXA4 can explain this differential effect (Chiang et al, 2006). Changes in cytoskeletal protein phosphorylation with consequent cell arrest occur in neutrophils exposed to LXA4, whilst mobilization of intracellular Ca^2+^ occurs in monocytes and macrophages, thus promoting chemotaxis. LXA4, at low nanomolar concentrations, generally decreases neutrophil activity, with lower levels of CD11b/CD18 expression, ROS formation and NFκB activity, as well as decreased synthesis of pro-inflammatory chemokines and cytokines. In monocytes and macrophages, LXA4 also promotes non-phlogistic phagocytosis of apoptotic neutrophils with reduced release of CXCL8 (Jozsef et al, 2002). Resolvins are another class of pro-resolving lipid mediators that were initially identified in exudates from mouse air-pouch models during the spontaneous resolution of inflammation. Resolvin E1 binds ChemR23 on monocytes, macrophages and dendritic cells to attenuate TNF-mediated NFκB activation, thus forming an anti-inflammatory signalling pathway (Arita et al, 2007). Furthermore, resolvin E1 reduces neutrophil infiltration in murine peritonitis and shortens the resolution interval while modifying the expression of miRNAs that target genes involved in resolution (Recchiuti et al, 2011). This effect appears to involve specific G protein-coupled receptors, namely ALX/FPR2 and GPR32 (Krishnamoorthy et al, 2012).

## Restoration of tissue functionality

The functional recovery of homeostasis after an inflammatory injury requires tissue repair and reestablishment of tissue functionality. The underlying mechanisms are complex and tissue-dependent but require a tight interplay between macrophages, stem and progenitor cells, together with stromal cells to prevent fibrosis or scar formation, a pathophysiological condition that leads to ineffective and inappropriate tissue function. Landmark studies have shown that macrophages orchestrate these reparative processes (Leibovich & Ross, 1975; Polverini et al, 1977). Recent work points towards a critical role of alternatively activated macrophages (Lucas et al, 2010; Saclier et al, 2013; Sindrilaru et al, 2011), which secrete anti-inflammatory and reparative mediators including IL1 receptor antagonist, IL10, TGF-β and VEGF. The generation of growth factors promotes cell proliferation and protein synthesis in neighbouring cells (Rappolee et al, 1988), while the production of proteases and their inhibitors regulate extracellular matrix (ECM) composition and remodelling. Macrophage-derived TGF-β contributes to tissue regeneration and wound repair by promoting (i) fibroblast differentiation into myofibroblasts, (ii) expression of tissue inhibitors of metalloproteinases (TIMPs) that regulate ECM remodelling and (iii) synthesis of interstitial fibrillar collagens by myofibroblasts. Macrophages also produce the majority of wound-associated VEGF, assuring angiogenesis and restoration of oxygen supply (Knighton et al, 1983). M2 macrophages also produce MMPs and TIMPs that control ECM turnover engulf and digest various ECM components that would promote tissue-damaging M1 macrophage responses (Atabai et al, 2009), and secrete specific chemokines that recruit fibroblasts and regulatory T (T_Reg_) cells (Curiel et al, 2004).

There is scattered evidence supporting the hypothesis that macrophages interact with progenitor or stem cells, and that this interplay may contribute to repair and remodelling. Mesenchymal stem cells (MSCs) are candidates for cellular therapies aimed at promoting tissue repair or immunoregulation (Uccelli et al, 2008). MSCs engage in a bidirectional interaction with cells of the macrophage lineage. M2-like macrophages and their mediators promote growth of human MSCs (Freytes et al, 2013) and can also stimulate MSC motility (Anton et al, 2012). Conversely, MSCs profoundly influence macrophage function by inducing a IL-10^high^ IL12^low^ alternative activation phenotype (Kim & Hematti, 2009). Injection of MSCs is associated with promotion of functional recovery after spinal cord injury, including axonal preservation and reduced scar formation (Nakajima et al, 2012). Neuroprotection in this system is attributed to MSC-induced shift in macrophage polarization from M1 to M2.

The fate of infiltrating macrophages is not fully understood. It has been postulated that after performing their central tasks in resolution, macrophages emigrate to the draining lymph node, where they may play an important role in the presentation of antigens from the inflamed site (Bellingan et al, 1996). MMP-mediated shedding of β_2_ integrin seems important for macrophage egress from the injured tissue (Cao et al, 2005; Gomez et al, 2012). A failure in macrophage egression results in accumulation and may potentially lead to chronic inflammatory diseases, such as atherosclerosis (Llodra et al, 2004).

## Suppressive immune cells as emerging players in resolution

**Figure 3:**
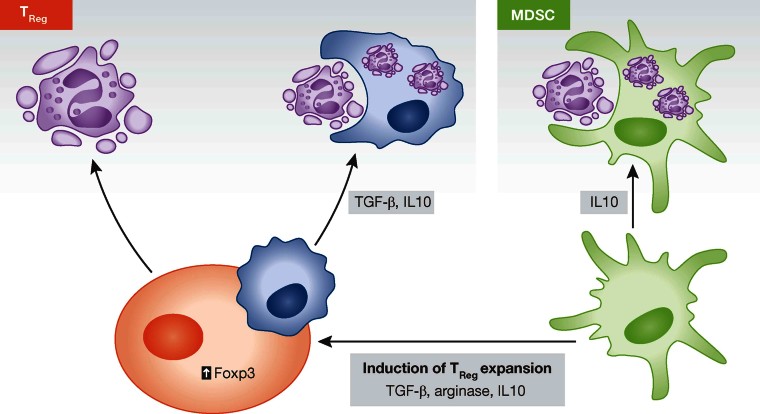
Role of accessory cells in resolution. MDSCs clear apoptotic neutrophils, release anti-inflammatory IL10 and contribute to the expansion of T_Reg_. T_Reg_ stimulate neutrophil apoptosis, enhance the efferocytosis capacity of macrophages and secrete resolving cytokines like IL10 and TGF-β in a contact-dependent manner.

Although neutrophils and macrophages have traditionally been looked upon as dominant cell types during the resolution phase, accessory cells such as myeloid-derived suppressor cells (MDSCs) and T_Reg_ have more recently emerged as important players during resolution and may link innate and adaptive immune systems (Fig 3).

T_Reg_, a subset of CD4^+^CD25^+^ T lymphocytes that co-express the transcription factor Foxp3 (Hori et al, 2003), regulate immune responses through the secretion of immunosuppressive and pro-resolving cytokines such as IL10 and TGF-β, thus being key players in immune homeostasis (Asseman et al, 1999; Fahlen et al, 2005). To date, beneficial roles of T_Reg_ have emerged in a number of inflammatory diseases including rheumatoid arthritis (Cao et al, 2003), multiple sclerosis (Viglietta et al, 2004) and atherosclerosis (Ait-Oufella et al, 2006). Traditionally, T_Reg_ are activated in an antigen-dependent manner to inhibit self-reactive or pathogenic T cells. However, T_Reg_ may also exert functions independent of inhibition of lymphocyte function, as was shown in a model of LPS-induced acute lung injury (D'Alessio et al, 2009). The transfer of wild type T_Reg_ to *Rag1*^−/−^ mice lacking mature T and B cells caused abrogation of lung inflammation, accompanied by increased apoptosis of alveolar neutrophils and macrophage efferocytosis, together with reduced pro-inflammatory cytokines and doubling of TGF-β levels. T_Reg_ abrogated TNF production and boosted TGF-β release in isolated alveolar macrophages in a contact-dependent manner. These results are in line with a previous study demonstrating that anti-inflammatory macrophages induce differentiation of T_Reg_ via TGF-β (Savage et al, 2008). These findings highlight the cross-talk between innate and adaptive immunity where cells of both systems regulate each other to coordinate resolution.

MDSCs represent a heterogenic population of immature myeloid cells expressing Gr1 and CD11b in mice and suppress T-cell functions (Bronte et al, 2000; Ribechini et al, 2010). In healthy individuals immature myeloid cells migrate to different peripheral organs, where they differentiate into dendritic cells, macrophages and/or granulocytes. However, under pathological conditions such as acute or chronic infection, sepsis, trauma or tumours, their differentiation is partially blocked leading to the generation and accumulation of MDSC. One of the original features of MDSCs is the production of arginase I (ARG1) or inducible nitric oxidase synthase (iNOS) that initiate the release of nitric oxide (NO) and ROS involved in programmed cell death or apoptosis and other immunosuppressive mechanisms (Gabrilovich & Nagaraj, 2009). The transcription factor STAT1 is of key importance in the signalling pathway leading to increased expression of ARG1 and iNOS. Indeed, MDSCs from *Stat1*^*−/−*^ mice fail to up-regulate these enzymes and therefore do not inhibit T-cell responses (Kusmartsev et al, 2005). In contrast to MDSCs, neutrophils create a pro-inflammatory environment upon activation that further activates T cells and can induce their differentiation by release of IL12, IL4 or IL6 (Muller et al, 2009).

MDSCs exert beneficial activities in inflammatory conditions such as sepsis, inflammatory bowel disease, autoimmune encephalomyelitis and multiple sclerosis (Delano et al, 2007; Haile et al, 2008; Zhu et al, 2007). MDSCs also phagocytose apoptotic neutrophils and produce large amounts of IL10 contributing to resolution in bacterial pneumonia (Poe et al, 2013). Interestingly, MDSCs induce expansion of T_Reg_ by increasing their Foxp3 expression in a IL10, TGF-β and arginase dependent manner (Huang et al, 2006; Serafini et al, 2008). Although research on the function of MDSCs in resolution is still in its infancy, the latter findings suggest that MDSCs might exert a regulatory role in resolution by establishing a link with the adaptive immune system.

**Box 1: Criteria for resolution-inducing mediators**

Resolution, the return to normal inflammatory conditions, does not merely consist in catabolism of inflammatory mediators and abrogation of inflammatory processes. Instead, resolution is an active and coordinated anti-inflammatory, pro-resolving programme aimed at restoration of tissue homeostasis, integrity and function. Pro-resolving mediators should ideally fulfill the following criteria:

1. Stop of inflammatory cell **recruitment**—the abrogation of neutrophil influx to block delivery of tissue-toxic proteases and oxygen radicals is of crucial importance in resolution.
2. Induction of neutrophil **apoptosis** and clearance (**efferocytosis**)—removal of apoptotic neutrophils is of dual importance: it induces reprogramming of macrophages and prevents spilling of potentially toxic contents from the neutrophil cytoplasm as they become necrotic.
3. **Egress** of immune cells—following efferocytosis, macrophages and dendritic cells leave the site of inflammation.
4. Positive modulation of the **immune response**—instruction of suppressive immune cells and the adaptive immune response to help dealing with subsequent encounters.
5. Induction of tissue **repair**—return to homeostasis without fibrosis or scar formation marks the final step of resolution.

**Table:** 

	↓Recruitment	↑Apoptosis/efferocytosis	↑Egress	↑Immune response	↑Tissue repair	Refs.
Annexin A1	+	+	N.D.	+	+	Perretti & D'Acquisto (2009)
Chemerin C15	+	+	N.D.	N.D.	N.D.	Cash et al (2008), Cash et al (2010)
Lipoxins	+	+	+	+	+	Serhan et al (2008)
Resolvins	+	+	+	No effect	+	Ariel & Serhan (2007)
Galectin-1	+	N.D.	N.D.	−	+	Ilarregui et al (2009)
Glucocorticoids	+	+	N.D.	−	−	Perretti & D'Acquisto (2009)
Adenosine	+	+	N.D.	N.D.	+	Csoka et al (2012), Koroskenyi et al (2011)
Melanocortins	+	+	N.D.	N.D.	+	Patel et al (2011)

+, stimulation; −, inhibition; N.D., not defined.

## Therapeutic stimulation of resolution

Agents that target pro-inflammation mediators have dominated drug research for inflammatory diseases for the last decades. Current anti-inflammatory therapies control cardinal signs of inflammation, mostly antagonizing specific pathways that are engaged when acute inflammation sets in. To transfer of concepts of resolution from bench to bedside requires a shift in emphasis from inhibitory therapy to replacement therapy, *i.e.* from antagonism to agonism. The advantage of immunoresolvents would be to limit continued neutrophil infiltration, counter-regulate pro-inflammatory mediators, enhance the containment and phagocytosis of cellular debris and apoptotic neutrophils, and promote restoration of tissue homeostasis (Box 1). Therapies that actively promote resolution may also have the advantage of enhancing innate immune responses to bacterial infections (Chiang et al, 2012), whereas established anti-inflammatory therapies such as anti-TNF strategies may be immunosuppressive (Bruns et al, 2009).

### Inhibition of leukocyte recruitment

The inhibition of continued leukocyte recruitment is essential to favour the return to homeostasis. However, the therapeutic targeting of cell adhesion molecules or chemokines to limit inflammatory cell recruitment has so far been largely unsuccessful. Pro-resolving AnxA1 dampens neutrophil tissue accumulation by several mechanisms including downregulation of transendothelial migration (Perretti et al, 1996), promotion of neutrophil apoptosis (Perretti & Solito, 2004), and stimulation of the removal of dead neutrophils (Scannell et al, 2007). The combination of these mechanisms results in potent pro-resolving effects in *in vivo* models of inflammation (Table 1; Dalli et al, 2008; Vago et al, 2012). Similar activities have also been ascribed to chemerin-derived peptides. While chemerin primarily attracts antigen-presenting cells, C-terminal peptides released from chemerin by cysteine proteases have opposite effects—they block neutrophil tissue infiltration and the release of pro-inflammatory mediators from classically activated macrophages (Cash et al, 2008). In addition, chemerin-derived peptides promote clearance of dead neutrophils by macrophages (Cash et al, 2010). Among the resolution-inducing agents, resolving lipid mediators like LXA4, resolvin E1 and D1, and protectins exert multiple pro-resolving effects, including inhibition of neutrophil tissue infiltration, induction of neutrophil apoptosis and efferocytosis and stimulation of tissue repair (Serhan et al, 2011). In animal models of inflammation, resolvin E1 is protective in a model of colitis as shown by decreased neutrophil tissue infiltration, pro-inflammatory gene expression and improved survival (Arita et al, 2005). Similarly, resolvin E1 reduces leukocyte infiltration in a mouse model of asthma and ultimately improves lung function (Haworth et al, 2008). Although LXA4 is equally potent in inducing resolution, due to rapid degradation in the blood stream, various delivery strategies have evolved, including the use of stable forms such as fluorinated analogues (Clish et al, 1999). Nanoparticles—efficient delivery microstructures—can be enriched with aspirin-triggered resolvin D1 or a LXA4 analogue to accelerate resolution in experimental peritonitis (Norling et al, 2011).

**Pending issues**

Integrated understanding of the complex interaction between pro- and anti-resolution mediators.
Existence of positive networks in resolution, where one pro-resolving mediator would induce another one.
Further investigation on the role of new cellular players in resolution.
Design of drugs integrating the key aspects that define a potent resolution-inducing mediator that incites resolution rather than simply buffering inflammation.
Discernment of tissue-specific resolution mechanisms.

### Induction of neutrophil apoptosis

Given the central role of neutrophil apoptosis in resolution, strategies aiming at the promotion of neutrophil apoptosis have emerged (Table 1; Geering & Simon, 2011). Despite being end-differentiated cells, the use of cyclin dependent kinase inhibitors such as R-roscovitine has been demonstrated to promote neutrophil apoptosis, leading to resolution in a wide range of inflammatory models (Gherardi et al, 2004; Rossi et al, 2006; Sekine et al, 2008; Zoja et al, 2007). However, a long-term treatment that induces neutrophil apoptosis may attenuate the innate immune response; hence molecules targeting the inflammation-induced prolongation of neutrophil life span are preferable. 5-epi-LXA4 induces resolution in a mouse model of lung injury by counteracting the myeloperoxidase-mediated suppression of neutrophil apoptosis (El Kebir et al, 2009). Ectoine, a compatible solute with anti-inflammatory properties, is able to prevent the anti-apoptotic mechanisms that extend neutrophil life span in an inflammatory microenvironment. Importantly, ectoine does not affect apoptosis of non-activated neutrophils (Sydlik et al, 2013). Finally, PD098059, an ERK inhibitor, was shown to counteract GM-CSF-induced neutrophil survival and to promote resolution in a rat model of carrageenan-induced pleurisy (Sawatzky et al, 2006). #box^2^

### Enhancement of efferocytosis and macrophage reprogramming

An increase of neutrophil apoptosis without a “disposal strategy” (*i.e.* efferocytosis) for the cell remnants perpetuates inflammation. AnxA1, resolvins and LXA4 were all shown to induce efferocytosis, which partially explains their resolution-inducing activities *in vivo* (Table 1). In addition, in a model of chronic granulomatous disease, IL4 administration induces alternative macrophage activation with enhanced ability to clear apoptotic neutrophils (Fernandez-Boyanapalli et al, 2009). A recent study used an alternative strategy to enhance the surface expression of *eat me* signals on apoptotic cells and ensure clearance; delivery of AnxA5-RGD complexes that bind to PS on apoptotic cells and on the vitronectin receptor on the efferocyte would promote cell clearance and secretion of IL-10 (Schutters et al, 2013). Beyond their use as vehicles to deliver resolving compounds (Metselaar et al, 2004), liposomes may also be used to mimic apoptotic cells to induce macrophage reprogramming. For instance, PS-containing liposomes can mimic the phagocyte stimulation achieved by apoptotic cells and induce secretion of pro-resolution mediators (Table 1). In addition, PS-liposomes are preferentially ingested by macrophages (Geelen et al, 2012), highlighting their potential in targeting macrophage-driven resolution mechanisms.

### Clinical trials of pro-resolving drugs

In addition to the pharmacological activities demonstrated in animal models, several on-going clinical trials are yielding positive results. Resolvin E1 (RX-10001) and a synthetic analogue (RX-100045) are being tested in numerous inflammatory diseases including dry eye, retinal disease, asthma, inflammatory bowel diseases, rheumatic arthritis and cardiovascular diseases. RX-100045 has successfully completed the phase II study in dry eye patients and is scheduled to enter a phase III randomized, placebo-controlled, multi-centre study (Lee, 2012). In another clinical study, a LXA4-based compound was tested for the topical treatment of eczema (Wu et al, 2013). Albeit in a small number of patients, the drug reduced the severity of eczema to a similar extent as steroid therapy in a double-blind placebo-controlled setting, thus indicating efficacy and safety of resolving mediators in a clinical study. AP214, a melanocortin agonist developed by Action Pharma AS (recently acquired by Abbott), elicits pro-resolving and tissue-protective effects in experimental systems (Montero-Melendez et al, 2011); in clinical settings, it effectively reduces acute kidney injury associated with major cardiac surgery. This study offers positive expectations as it has presented phase 2b top-line results in the evaluation of the efficacy, safety and tolerability of the compound. A second phase 2b study is scheduled for the end of 2012.

## Conclusion

Our understanding of resolution as an active process has come a long way since the early observations by Elie Metchnikoff who observed that neutrophils are phagocytosed by macrophages and how this clearance resolves tissue inflammation (Mechnikov, 1988). Resolution is now looked upon as a complex process where apoptosis of neutrophils and their subsequent clearance herald potent anti-inflammatory, tissue-restoring mechanisms. To fully appreciate the complexity of such processes and to design resolution-based therapeutic strategies, future work is needed to discern differences in the mechanisms of resolution in acute and chronic inflammatory disorders as well as decipher the tissue-specific resolution networks.

## For more information

Information about clinical trials:

http://clinicaltrials.gov/

Information about chemokine receptor nomenclature:

http://www.iuphar-db.org

Information about the authors:

http://www.phagocytes.net/groups/soehnlein.html

http://www.whri.qmul.ac.uk/staff-all/staff-research/207-perretti-mauro

## Abbreviations

Annexin A1: A glucocorticoid-regulated protein, highly abundant in myeloid cells such as neutrophils and macrophages, with profound effects on several phases of the resolution of inflammation.
Chemokines: Low molecular weight cytokines with chemotactic effects on leukocytes.
Decoy receptor: Receptor for cytokines that, while irreversibly binding to its ligands, does not trigger activation of the corresponding intracellular signalling pathway, hence acting as a scavenger of these molecules.
Efferocytosis: Phagocytosis and clearance of an apoptotic cell.
Find me/eat me signals: Group of molecules secreted or exposed at the surface of an apoptotic cell that guide efferocytes to facilitate their disposal.
Liposome: Spherical particle composed by a lipid bilayer, containing a lumen with the capacity to encapsulate and transport substances in biological systems, thus being a suitable vehicle for drug delivery.
Myeloid derived suppressor cells: Heterogeneous population of early myeloid progenitors with the ability to suppress T cells activity.
Pro-resolving lipid mediators: Bioactive lipids secreted in response to an inflammatory state that stimulate molecular and cellular events to achieve resolution.
Resolution-phase macrophage: Macrophage that, in response to stimuli announcing the conclusion of an acute inflammation, reprograms its phenotype and acts towards the termination of the inflammatory process and return to homeostasis.
T_Reg_: Naturally immunosuppressive subpopulation of CD4^+^ T cells characterized by constitutive expression of the transcription factor Foxp3.
